# Immune mediators as predictive biomarkers for anti-PD-1 antibody therapy in urothelial carcinoma

**DOI:** 10.3389/fphar.2023.1269935

**Published:** 2023-11-13

**Authors:** Yosuke Shibata, Takeshi Kishida, Taku Kouro, Feifei Wei, Yuka Igarashi, Hidetomo Himuro, Takeaki Noguchi, Mitsuyuki Koizumi, Takahisa Suzuki, Kimito Osaka, Yusuke Saigusa, Tetsuro Sasada

**Affiliations:** ^1^ Department of Urology, Kanagawa Cancer Center, Yokohama, Kanagawa, Japan; ^2^ Division of Cancer Immunotherapy, Kanagawa Cancer Center Research Institute, Yokohama, Kanagawa, Japan; ^3^ Cancer Vaccine and Immunotherapy Center, Kanagawa Cancer Center, Yokohama, Kanagawa, Japan; ^4^ Department of Biostatistics, School of Medicine, Yokohama City University, Yokohama, Kanagawa, Japan

**Keywords:** immune checkpoint inhibitors, anti-PD-1 antibody, pembrolizumab, cytokine, chemokine, immune-related adverse event

## Abstract

**Introduction:** This study aimed to identify immune mediators, including cytokines, chemokines, and growth factors, in the plasma for predicting treatment efficacy and immune-related adverse events (irAEs) in advanced urothelial carcinoma (aUC) treated with immune checkpoint inhibitors (ICIs).

**Methods:** We enrolled 57 patients with aUC who were treated with the anti-programmed cell death protein 1 (PD-1) antibody pembrolizumab after the failure of platinum-based chemotherapy between February 2018 and December 2020. Plasma levels of 73 soluble immune mediators were measured before and 6 weeks after initiating pembrolizumab therapy. The association of estimated soluble immune mediators with clinical outcomes, including overall survival (OS), progression-free survival (PFS), anti-tumor responses, and irAEs, were statistically evaluated.

**Results:** In the multivariate analysis, levels of 18 factors at baseline and 12 factors during treatment were significantly associated with OS. Regarding PFS, baseline levels of 17 factors were significantly associated with PFS. Higher levels of interleukin (IL)-6, IL-8, soluble tumor necrosis factor receptor 1 (sTNF-R1), and IL-12 (p40), both at baseline and post-treatment, were significantly associated with worse OS. Conversely, low IL-6 and high TWEAK levels at baseline were associated with irAEs. Among identified factors, interferon (IFN) γ and IL-12 (p40) were repeatedly identified; high baseline levels of these factors were risk factors for worse OS and PFS, as well as progressive disease. Notably, using correlation and principal component analysis, factors significantly associated with clinical outcomes were broadly classified into three groups exhibiting similar expression patterns.

**Discussion:** Measuring plasma levels of soluble immune mediators, such as IL-6, IL-8, sTNF-R1, IFNγ, and IL-12 (p40), could be recommended for predicting prognosis and irAEs in ICI-treated patients with aUC.

## Introduction

Platinum-based chemotherapy is the most commonly used first-line therapy for advanced urothelial carcinoma (aUC). Following the failure of first-line regimens, pembrolizumab, a humanised monoclonal antibody targeting programmed death protein-1 (PD-1), was approved for second-line treatment as the first immune checkpoint inhibitor (ICI) based on the KEYNOTE-045 trial (Bellmunt et al., 2017). In 2021, maintenance therapy with avelumab was approved for patients who responded to platinum-based chemotherapy based on the findings of the JAVELIN Bladder 100 trial (Powles et al., 2020). Pembrolizumab, the most commonly used ICI for aUC, has shown a response rate of 21.1%, and 68% of patients with a good response to pembrolizumab were found to exhibit a long-term response exceeding 1 year (Bellmunt et al., 2017). Conversely, 60.9% of patients reportedly experienced immune-related adverse events (irAEs), 15% of which were ≥ grade 3 severe irAEs, thereby resulting in treatment discontinuation in 5.6% of patients (Bellmunt et al., 2017).

Currently, only a limited number of patients can receive third-line therapy following disease progression after second-line treatment with ICIs. The antibody-drug conjugates, enfortumab vedotin and sacituzumab govitecan, and the fibroblast growth factor receptor inhibitor, erdafitinib, have recently demonstrated efficacy as third-line treatments after ICI therapy (Tagawa et al., 2021; Yu et al., 2021; Siefker-Radtke et al., 2022). Thus, given that switching to subsequent treatment remains urgent in patients who fail to respond to pembrolizumab, biomarkers to predict the clinical effects before or at early time points of ICI treatment need to be developed.

In other cancers, such as lung cancer, the expression of programmed death ligand 1 (PD-L1) has been clinically employed as a biomarker for ICIs to predict their efficacy. However, in urothelial carcinoma (UC), the KEYNOTE-045 and JAVELIN Bladder 100 trials found no clinical significance for PD-L1 expression (Bellmunt et al., 2017; Powles et al., 2020). In the current study, we analyzed soluble immune mediators in the plasma of patients with aUC treated with pembrolizumab to determine their clinical roles in predicting ICI efficacy and irAEs.

## Materials and methods

### Patients

Herein, we enrolled 57 patients initiated on the anti-PD-1 antibody pembrolizumab after platinum-based chemotherapy for aUC at Kanagawa Cancer Center between February 2018 and December 2020. Peripheral blood (heparin anticoagulated) was collected from these patients at pembrolizumab treatment initiation and 6 weeks after initiation to measure soluble immune mediators. All eligible patients received pembrolizumab (200 mg/body weight) every 3 weeks. The studies involving human participants were reviewed and approved by the Institutional Review Boards of Kanagawa Cancer Center. The patients/participants provided their written informed consent to participate in this study.

### Measurement of soluble immune mediators in plasma

Before and after pembrolizumab administration, a bead-based multiplex assay (Bio-Plex 200 system; Bio-Rad Laboratories, Hercules, CA) was used to evaluate the plasma levels of soluble immune mediators, including cytokines, chemokines, and growth factors. As the plasma amounts were limited, we used two multiplex kits (Bio-Plex Pro™ Human Chemokine Panel 40-Plex kit and Human Inflammation Panel 1, 37-Plex kit; Bio-Rad Laboratories), which could measure many different immune mediators in a single well at the same time, in accordance with the manufacturer’s instructions. The kits contained the reagents to comprehensively measure 73 soluble immune mediators that may be important for antitumor immunity and inflammation: interleukin (IL)-1β, IL-2, IL-4, IL-6, IL-8, IL-10, IL-11, IL-12 (p40), IL-12 (p70), IL-16, IL-19, IL-20, IL-22, IL-26, IL-27, IL-28A, IL-29, IL-32, IL-34, IL-35, interferon (IFN) α2, IFNβ, IFNγ, tumor necrosis factor (TNF) α, granulocyte-macrophage colony-stimulating factor, C-C motif chemokine ligand (CCL) 1, CCL2, CCL3, CCL7, CCL8, CCL11, CCL13, CCL15, CCL17, CCL19, CCL20, CCL21, CCL22, CCL23, CCL24, CCL25, CCL26, CCL27, C-X-C motif chemokine ligand (CXCL) 1, CXCL2, CXCL5, CXCL6, CXCL9, CXCL10, CXCL11, CXCL12, CXCL13, CXCL16, CX3CL1, macrophage migration inhibitory factor, sCD30, sCD163, chitinase 3-like-1, gp130, IL-6Rα, soluble TNF (sTNF)-R1, sTNF-R2, APRIL, BAFF, LIGHT, pentraxin-3, TSLP, TWEAK, osteocalcin, osteopontin, matrix metalloproteinase (MMP)-1, MMP-2, and MMP-3.

In brief, patients’ plasma stored at −80°C was thawed at 4°C overnight and was diluted four times with sample diluent buffer. The diluted plasma (50 μL) was incubated with antibody-coupled beads in the filter plate (Multiscreen HTS BV, 1.2 μm, Merck Millipore, Burlington, MA) for 1 h at room temperature on plate mixer. Then, the plate was set on the vacuum manifold and beads were washed with 100 μL of wash buffer three times and incubated with biotinylated detection antibody cocktail for 30 min at room temperature on the plate mixer. The beads were then washed three times with wash buffer and incubated with streptavidin-phycoerythrin for 10 min at room temperature on a plate shaker. After three washes, beads were resuspended in 150 μL of assay buffer and subjected to analysis on the Bio-Plex 200 system. The fluorescence intensity values obtained were plotted against standard curves generated from standard cocktail measurements to calculate the concentrations of soluble immune mediators. All assays were performed on the same day using reagent kits and standards manufactured from the same lot.

### Statistical analysis

Tumor response was assessed according to the Response Evaluation Criteria in Solid Tumours, version 1.1. Adverse events were evaluated according to the Common Terminology Criteria for Adverse Events, version 5.0. Two analyses were performed: the levels of soluble immune mediators before treatment (baseline analysis) and potential alterations after 6 weeks of treatment (change analysis). Overall survival (OS) was calculated from the start date of pembrolizumab therapy to the date of death or last follow-up. Patients who were alive were censored at the date of the last contact. Progression-free survival (PFS) was calculated from the start date of pembrolizumab therapy to the date of progression, death, or last follow-up. OS and PFS were evaluated using Cox proportional hazard regression models and log-rank tests. Logistic regression analysis was used to estimate the odds ratios for progressive disease (PD) and irAEs. In the baseline analysis with Cox proportional hazard regression models, continuous measurements of baseline soluble immune mediator levels were approximately log-normally distributed and analysed as log-10 transformed. In the baseline analysis with the log-rank test, patients were classified into “high” and “low” groups by setting the median as the cut-off value. To analyze the altered levels, patients were divided into two groups: those with decreased levels and those with unchanged or increased levels after pembrolizumab administration.

For multivariate analysis of OS, the following clinical factors known to be significantly associated with OS were included in the model: Eastern Cooperative Oncology Group performance status (ECOG PS) and liver metastases (Yanagisawa et al., 2022). For multivariate analysis of PFS, PD, and irAE, variables were selected using the forward stepwise method using Bayesian information criterion from the following clinical factors: age, sex, ECOG PS, liver metastasis, lung metastasis, bone metastasis, lymph node metastasis only, radical surgical history, pathological grade, neutrophil-to-lymphocyte ratio, platelet count, hemoglobin, albumin, and irAE history. Each soluble immune mediator was used in multivariate analysis. All these clinical factors could have been better included in the multivariate model; however, the small number of events limited the number of clinical factors that could be included in the model. The mediators were characterized using principal component analysis. The correlations between each mediator were evaluated using Spearman’s rank correlation coefficient analysis. All *p*-values were two-sided, with a significance level set at 0.05. Statistical analyses were performed using EZR (version 3.5.2; https://www.jichi.ac.jp/saitama-sct/SaitamaHP.files/statmedEN.html) and R (version 4.1.3; https://www.r-project.org/). The network was visualised using the Gephi software (Ver 0.9.1; https://gephi.org/).

## Results

### Patient characteristics

Table 1 presents the patient’s background. The median follow-up was 7.2 months (range: 0.2–34.8), and the median age at pembrolizumab initiation was 73 years (range: 44–85). Primary cancer sites were bladder in 23 (40.4%), ureter in 18 (31.6%), and renal pelvis in 16 patients (28.1%). Histologic types included UC in 50 patients (87.7%), squamous cell variant in 5 patients (8.8%), and sarcomatoid variant or micropapillary variant in 1 patient (1.8%) each. Seventeen patients (29.8%) exhibited only lymph node metastasis. Of the 39 patients with visceral metastases, 12 (21.1%), 21 (36.8%), and 14 (24.6%) presented liver, lung, and bone metastases, respectively.

**TABLE 1:** Baseline patient characteristics and treatment received.

Characteristic		No. (%)
Total No.		57
Follow-up period, median (range), months		7.2 (0.2–34.8)
Age, median (range), year		73 (44–85)
Sex		
	Male	43 (75.4)
	Female	14 (24.6)
ECOG PS		
	0–1	40 (70.2)
	≧2	17 (29.8)
Primary lesion		
	Renal pelvis	16 (28.1)
	Ureter	18 (31.6)
	Bladder	23 (40.4)
Radical Surgery		
	Done	37 (64.9)
	Not done	20 (35.1)
Histology		
	Urothelium	50 (87.7)
	Squamous	5 (8.8)
	Sarcomatoid	1 (1.8)
	Micropapillary	1 (1.8)
Pathological Grade		
	1–2	8 (14.0)
	3	38 (66.7)
	Unknown	11 (19.3)
Site of metastases		
	Lymph node only	17 (29.8)
	Liver	12 (21.1)
	Lung	21 (36.8)
	Bone	14 (24.6)
Systemic therapy prior to pembrolizumab administration		
	Gemcitabine/cisplatin	51 (89.5)
	Gemcitabine/carboplatin	8 (14.0)
	Methotrexate/vinblastine/doxorubicin/cisplatin	3 (5.3)
	Paclitaxel/carboplatin	3 (5.3)
	Paclitaxel/ifosfamide/nedaplatin	3 (5.3)
	Gemcitabine/nedaplatin	2 (3.5)
	Durvalumab/gemcitabine/cisplatin	1 (1.8)
	Avelumab	1 (1.8)
No. of chemotherapy lines prior to pembrolizumab		
	1	48 (84.2)
	2	5 (8.8)
	≧3	4 (7.0)
Best response to Pembrolizumab		
	CR	4 (7.0)
	PR	19 (33.3)
	SD	4 (7.0)
	PD	30 (52.6)
irAE		
	None	25 (43.9)
	G1-2	20 (35.1)
	≧G3	12 (21.1)

Abbreviations: CR, complete response; ECOG PS, Eastern cooperative oncology group performance status; PD, progressive disease; PR, partial response; SD, stable disease; irAE, immune-related adverse event.

All patients were treated with pembrolizumab after one or more platinum-based chemotherapy regimens. Two patients received ICI treatment prior to pembrolizumab. One received durvalumab plus platinum-based chemotherapy, and the other received avelumab as maintenance therapy after platinum-based chemotherapy. At the time of analysis, 7 patients continued pembrolizumab; however, 48 patients discontinued pembrolizumab owing to treatment failure. In addition, two patients, one with grade 3 hypothyroidism and grade 2 myositis and another with grade 3 arthritis, discontinued pembrolizumab because of irAEs. The median OS and PFS were 12.6 [95% confidence interval (CI) 5.1–17.3] and 3.0 (95% CI 1.9–5.9) months, respectively. The objective response rate of pembrolizumab was 40.4% (23/57), while 4 (7.0%) patients exhibited complete response (CR), 19 (33.3%) had a partial response (PR), 4 (7.0%) experienced stable disease (SD), and 30 (52.6%) showed PD. Notably, two patients who discontinued pembrolizumab owing to irAEs achieved CR and PR.

### Association of soluble immune mediators and clinical factors with OS

ECOG PS and liver metastasis, which are frequently associated with OS, were selected and included in the multivariate analysis models; each soluble immune mediator was included in this model (Table 2). Considering the multivariate analysis, 18 mediators [IL-6, IL-8, IL-12 (p40), IFNγ, CCL1, CCL3, CCL7, CCL20, CXCL1, CXCL6, CXCL13, sCD30, sCD163, IL-6Rα, sTNF-R1, sTNF-R2, LIGHT, MMP-1] in the baseline analysis and 12 mediators [IL-6, IL-8, IL-12 (p40), IL-16, IL-22, IL-27, IL-28A, CCL21, CCL22, CXCL16, sTNF-R1, osteocalcin] in the change analysis were significantly associated with OS (Table 2).

**TABLE 2:** Cox multivariate analysis of factors associated with overall survival.

	Multivariate analysis		
Variable	HR	95% CI	*p*-value
Baseline analysis			
IL-6	5.40	1.82–16.02	0.002
IL-8	4.11	1.22–13.79	0.022
IL-12 (p40)	9.58	1.12–81.81	0.038
IFNγ	5.36	1.27–22.74	0.022
CCL1	1.12 × 10^2^	1.82–6.90 × 10^3^	0.024
CCL3	24.21	1.88–3.12 × 10^2^	0.014
CCL7	1.86	1.05–3.26	0.032
CCL20	5.19	1.29–20.81	0.020
CXCL1	4.56 × 10^2^	7.46–2.79 × 10^4^	0.003
CXCL6	2.42	1.12–5.23	0.024
CXCL13	3.23	1.08–9.62	0.035
sCD30	23.77	3.30–1.71 × 10^2^	0.001
sCD163	6.50	1.13–37.21	0.035
IL-6Rα	0.05	0.002–0.96	0.047
sTNF-R1	10.83	2.55–46.09	0.001
sTNF-R2	9.07	1.93–42.74	0.005
LIGHT	2.22	1.06–4.65	0.034
MMP-1	4.00	1.65–9.69	0.002
Change analysis			
IL-6	2.95	1.19–7.31	0.020
IL-8	7.72	2.38–25.06	<0.001
IL-12 (p40)	3.39	1.12–9.64	0.022
IL-16	4.91	1.12–21.62	0.035
IL-22	2.67	1.19–6.00	0.017
IL-27	0.35	0.15–0.83	0.016
IL-28A	3.63	1.41–9.35	0.007
CCL21	0.31	0.11–0.84	0.020
CCL22	0.43	0.19–0.98	0.045
CXCL16	3.01	1.17–7.76	0.022
sTNF-R1	3.75	1.10–12.86	0.035
Osteocalcin	0.34	0.13–0.92	0.033

In the baseline analysis, continuous measurements of mediator levels were log-10 transformed. In the change analysis, mediator levels were divided into two groups: those with decreased levels and those with unchanged or increased levels after pembrolizumab administration. ECOG PS, liver metastasis, and each mediator were modelled as three variables for multivariate analysis. The hazard ratios (HR) for ECOG PS and liver metastasis in this table are omitted. Abbreviations: CCL, C-C motif chemokine ligand; CXCL, C-X-C motif chemokine ligand; CI, confidence interval; IL, interleukin; IFNγ, interferon γ; MMP-1, matrix metalloproteinase-1; sTNF-R1, soluble tumor necrosis factor receptor 1.

**FIGURE 1:**
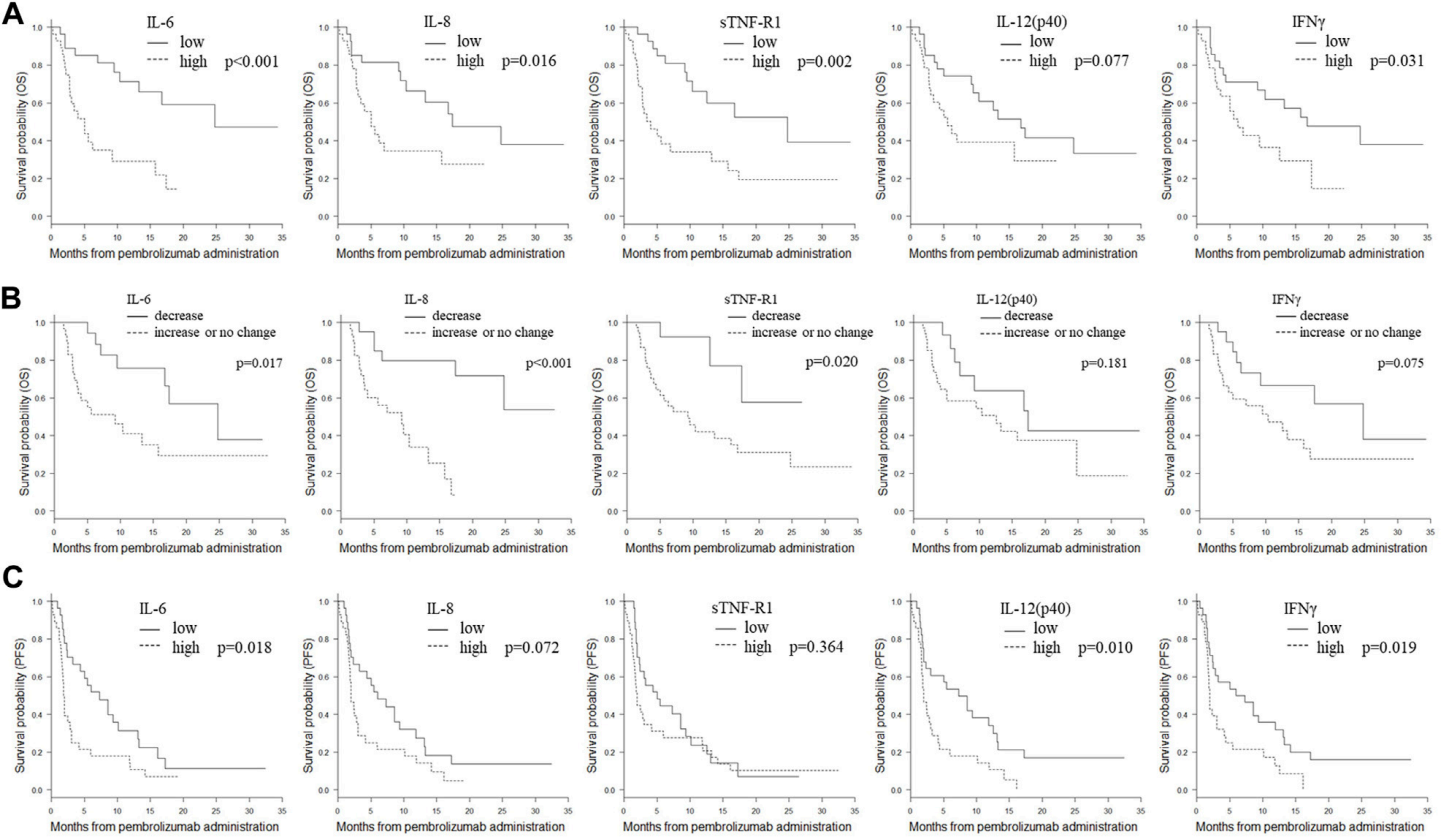
Kaplan-Meier plots of OS or PFS in the subgroups stratified by IL-6, IL-8, sTNFR-1, IL-12 (p40), and IFNγ. **(A)** Kaplan-Meier plots of OS in subgroups stratified by baseline plasma levels of IL-6, IL-8, sTNFR-1, IL-12 (p40), and IFNγ. The cut-off values between the high and low groups were the median values. *p*-values (log-rank test) are shown. **(B)** Kaplan-Meier plots of OS in the subgroups stratified by changes in plasma IL-6, IL-8, sTNFR-1, IL-12 (p40), and IFNγ after treatment. Patients were divided into two groups: those with decreased levels and those with unchanged or increased levels after treatment. *p*-values (log-rank test) are shown. **(C)** Kaplan-Meier plots of PFS in subgroups stratified by plasma levels of IL-6, IL-8, sTNFR-1, IL-12 (p40), and IFNγ after treatment. The cut-off values between the high and low groups were the median values. *p*-values (log-rank test) are shown. Abbreviations: IL, interleukin; IFNγ, interferon γ; OS, overall survival; PFS, progression-free survival; sTNF-R1, soluble tumor necrosis factor receptor 1.

Notably, IL-6, IL-8, IL-12 (p40), and sTNF-R1 were significantly associated with OS both at baseline and during treatment (baseline/change; *p* = 0.002/0.020, *p* = 0.022/<0.001, *p* = 0.038/0.022, and *p* = 0.001/0.035, respectively). High baseline levels of these factors and increased levels after treatment were significantly associated with worse OS. Subgroups stratified by these mediators were subjected to Kaplan-Meier analysis of OS (Figure 1). High baseline levels and increased levels after treatment of IL-6, IL-8, and sTNF-R1, but not those of IL-12 (p40), were significantly associated with worse OS (baseline/change; *p* < 0.001/0.017, *p* = 0.016/<0.001, *p* = 0.002/0.020, and *p* = 0.077/0.181, respectively; by log-rank analysis).

### Association of soluble immune mediators and clinical factors with PFS and PD

For PFS analysis, liver metastasis and platelet count were selected using the stepwise method and adapted to the multivariate model, with each mediator forced into this model (Table 3). Multivariate analysis revealed that 17 mediators [IL-2, IL-12 (p40), IL-29, IL-32, IL-35, IFNα2, IFNγ, CCL8, CCL15, CCL23, CXCL5, CXCL11, CXCL16, sCD30, LIGHT, TSLP, and MMP-2] in the baseline analysis were significantly associated with PFS.

**TABLE 3:** Cox multivariate analysis of PFS and multivariate logistic regression analysis of progressive disease.

Baseline analysis in PFS			
Variable	HR	95% CI	*p*-value
IL-2	5.15	1.17–22.62	0.030
IL-12 (p40)	9.23	1.76–48.32	0.008
IL-29	2.09	1.20–3.64	0.008
IL-32	2.96	1.13–7.75	0.026
IL-35	1.88	1.07–3.32	0.029
IFNα2	5.95	1.58–22.46	0.008
IFNγ	4.19	1.22–14.36	0.022
CCL8	0.04	0.003–0.35	0.004
CCL15	6.73	1.19–38.11	0.031
CCL23	2.40	1.09–5.29	0.030
CXCL5	1.49	1.03–2.16	0.032
CXCL11	0.17	0.03–0.79	0.024
CXCL16	0.14	0.02–0.90	0.038
sCD30	6.21	1.27–30.3	0.024
LIGHT	2.74	1.36–5.55	0.004
TSLP	8.26	1.12–60.77	0.038
MMP-2	6.39	1.07–38.01	0.041

Baseline analysis in PD			
Variable	OR	95% CI	*p*-value
IL11	3.48 × 10^3^	2.11–5.75 × 10⁶	0.031
IL12 (p40)	3.55 × 10^2^	1.54–8.18 × 10⁴	0.034
IFN-γ	1.22 × 10^2^	1.15–1.30 × 10⁴	0.043
CXCL13	28.00	1.64–4.85 × 10³	0.021
APRIL	2.57 × 10^2^	1.34–4.96 × 10⁴	0.038
TSLP	1.36 × 10^3^	1.80–1.03 × 10⁶	0.032
Osteopontin	24.00	1.24–4.49 × 10⁴	0.041

In the baseline analysis, continuous measurements of mediator levels were log-10 transformed. Liver metastasis, platelet count, and each mediator were modelled as three variables for the Cox multivariate analysis of PFS. The ECOG PS, liver metastasis, bone metastasis, and pathological grade were modelled as five variables for multivariate logistic regression analysis in PD. The hazard and odds ratios for the clinical factors in this table are omitted. Abbreviations: CCL, C-C motif chemokine ligand; CXCL, C-X-C motif chemokine ligand; CI, confidence interval; HR, hazard ratio; IL, interleukin; IFN, interferon; MMP-2, matrix metalloproteinase-2; OR, odds ratio; PD, progressive disease; PFS, progression-free survival.

For PD analysis, ECOG PS, liver metastasis, bone metastasis, and pathological grade were selected using the stepwise method and adapted to the multivariate model; each mediator was forced into this model (Table 3). In multivariate analysis, seven mediators [IL-11, IL-12 (p40), IFNγ, CXCL13, APRIL, TSLP, and osteopontin] in the baseline analysis were significantly associated with PD.

Notably, high baseline levels of IL-12 (p40) and IFNγ, which showed a significant association with PD, were also significantly associated with worse PFS and OS (PD/PFS/OS; *p* = 0.034, 0.008, 0.038, and *p* = 0.043, 0.022, and 0.022, respectively). Kaplan-Meier analysis of OS and PFS for IL-12 (p40) and IFNγ was performed (Figure 1). In the baseline analysis, low expression levels of IL-12 (p40) and IFNγ were significantly associated with prolonged PFS (*p* = 0.010 and *p* = 0.018, respectively). In the baseline analysis, low IFNγ expression was significantly associated with prolonged OS (*p* = 0.031); however, no association was detected in the change analysis (*p* = 0.075).

### Association of soluble immune mediators and clinical factors with irAEs

IrAEs were documented in 32 patients (56.1%), with ≥ grade 3 noted in 12 patients (21.1%); however, there were no deaths attributed to adverse events (Table 4). For multivariate analysis, liver metastasis was selected using a stepwise method and included in the model. When each of the mediators at baseline was forced into the model, low IL-6 (OR 0.182, 95% CI 0.03–0.88, *p* = 0.034) and high TWEAK (OR 350, 95% CI 3.62–33900, *p* = 0.012) levels at baseline were associated with irAEs.

**TABLE 4:** Immune-related adverse events of pembrolizumab (according to grade).

Toxicity	Grade1	Grade2	Grade3	Grade4	% of ≧Grade3
Adrenal insufficiency	0	0	2	0	3.50%
Hypothyroidism	2	3	1	0	1.75%
Hyponatremia	0	0	1	1	3.50%
Pneumonitis	0	0	1	0	1.75%
Diarrhea	3	0	3	0	5.26%
Anorexia	1	1	0	0	0
Malaise	0	2	0	0	0
Creatinine increased	1	0	1	0	1.75%
Hepatic failure	0	0	0	1	1.75%
Fever	3	1	0	0	0
Arthritis	0	0	1	0	1.75%
Skin disorders	10	3	0	0	0
Myositis	0	2	0	0	0
Hand-foot syndrome	1	0	0	0	0
Neuropathy	1	0	0	0	0
Edema	1	0	0	0	0
Dysgeusia	1	0	0	0	0

### Classification of identified mediators

**FIGURE 2:**
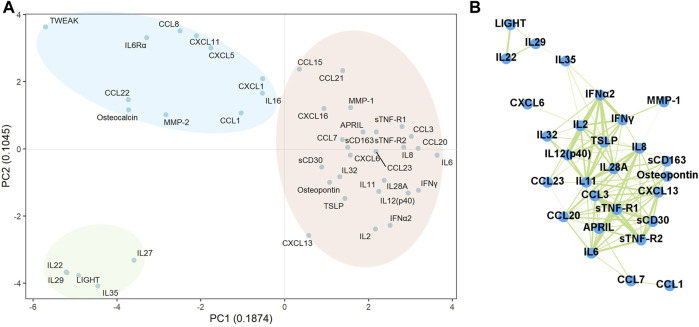
Classification of the plasma immune mediators. **(A)** The plasma immune mediators significantly associated with clinical outcomes, including OS, PFS, PD, and irAEs, were classified into three groups by principal component analysis. **(B)** The correlation among the plasma immune mediators significantly associated with clinical outcomes was examined using Spearman’s rank correlation coefficient analysis. Strong correlations (r > 0.5, *p* < 0.05; 118 pairs) were visualised using the weighted network, with the breadth of the line proportional to the correlation coefficient (from 0.50 to 0.84).

Using principal component analysis, we characterised identified mediators significantly associated with clinical outcomes, including OS, PFS, and irAEs. As shown in Figure 2A, these mediators were broadly classified into three groups. The first group contained five mediators, including IL-22, IL-27, IL-29, IL-35, and LIGHT, whereas the second group contained 11 factors, including IL-16, CCL1, CCL8, CCL22, CXCL1, CXCL5, CXCL11, IL-6Rα, TWEAK, osteocalcin, and MMP-2. The third group comprised 26 factors: IL-2, IL-6, IL-8, IL-11, IL-12 (p40), IL-28A, IL-32, IFNα2, IFNγ, CCL3, CCL7, CCL15, CCL20, CCL21, CCL23, CXCL6, CXCL13, CXCL16, sCD30, sCD163, sTNF-R1, sTNF-R2, APRIL, TSLP, osteopontin, and MMP-1. Notably, most mediators listed in the third group were highly correlated with each other (r > 0.5, *p* < 0.05, Spearman’s rank correlation coefficient analysis), as shown in Figure 2B.

## Discussion

In patients with aUC who fail to respond to ICI as second-line therapy, the OS was found to be 3.1 months, and only one-third of these patients can proceed to third-line therapy after ICI treatment failure (Gómez de Liaño Lista et al., 2020) mainly because of the rapid aUC progression and/or physical exhaustion caused by preceding treatments. Accordingly, several patients with aUC without indication of third-line treatment are compelled to undergo the best supportive care (BSC). Notably, the OS of patients who received third-line therapy after ICI treatment was 8.3 months, whereas that of patients with BSC was markedly brief, limited to approximately 1.5 months (Gómez de Liaño Lista et al., 2020). In addition, enfortumabu vedotin, acituzumab govitecan, and erdafitinib were recently approved as a promising therapy after ICI failure for aUC (Tagawa et al., 2021; Yu et al., 2021; Siefker-Radtke et al., 2022). Therefore, the development of valuable biomarkers for predicting the effects of ICI and the occurrence of irAEs before or during the early stages of treatment is urgently needed.

The Food and Drug Administration has approved PD-L1 expression, microsatellite instability/mismatch repair deficiency, and tumor mutational burden as predictive biomarkers of ICI (Twomey and Zhang, 2021). Although PD-L1 expression might be a predictive biomarker for ICI based on the findings of some previous trials on UC, the methods and results of PD-L1 assessment in each trial were different and inconsistent across trials. Integrated biomarkers that combine multiple factors have been shown to afford better results than a single Food and Drug Administration-approved predictive biomarker (Wang et al., 2021). Based on these results, a multifaceted approach may be necessary to precisely predict ICI efficacy. Although limited UC studies are available, comprehensive measurement of soluble immune mediators in plasma may be a promising strategy (Lim and Yoon, 2021).

In the present study, multivariate models demonstrated that baseline levels or post-treatment changes of several different immune mediators in plasma were significantly associated with OS and/or PFS in patients with aUC receiving pembrolizumab therapy. The principal component analysis demonstrated that these mediators, which were significantly associated with clinical outcomes, could be broadly classified into three groups. Interestingly, mediators that correlated with OS were mainly classified in the third group, whereas those correlated with PFS were more frequent in the second group. In addition, several examined factors were highly correlated with each other, although the precise mechanisms responsible for such a correlation remain unknown. Given that plasma immune mediators with distinct functions displayed similar expression patterns, it can be suggested that multiple factors, rather than a single factor, might affect the immunological tumor microenvironment concurrently and could be associated with clinical outcomes in patients treated with ICI. In-depth investigations assessing the functions and interactions of these mediators in ICI treatment would potentially reveal a more accurate prediction of ICI efficacy and aid in developing new treatment approaches.

It should be noted that both high baseline levels and elevated post-treatment levels of IL-6, IL-8, and sTNFR-1 were significantly associated with worse OS. IL-6 is involved in immunosuppression via various mechanisms. For example, IL-6-mediated STAT3 activation promotes the production of immunosuppressive molecules, such as vascular endothelial growth factor and arginase, and induces immunosuppressive cells, such as myeloid-derived suppressor cells (MDSCs) (Zhou et al., 2017), which reportedly serve as biomarkers of OS in patients with melanoma treated with ICI (Meyer et al., 2014). In addition, IL-6 limits anti-tumor T-cell responses by promoting the differentiation of M2 macrophages or acting on dendritic cells to inhibit the expression of MHC-II, CD80/86, and IL-12 and promote IL-10 production (Zhou et al., 2017). Furthermore, IL-6 activates MMP and forms desmoplastic tumor stroma (Dechow et al., 2004), which acts as a barrier to avoid recognition by the immune system and subsequent T-cell infiltration (Salmon and Donnadieu, 2012). Although higher IL-6 levels are reportedly associated with worse OS and PFS in patients with melanoma and non-small-cell lung carcinoma undergoing ICI (Keegan et al., 2020; Laino et al., 2020), reports on patients with UC are lacking. IL-8, a proinflammatory chemokine, inhibits anti-tumor immune responses by inducing MDSCs in the tumor microenvironment and expanding the desmoplastic tumor stroma, similar to IL-6 (Alfaro et al., 2016). In addition, IL-8 promotes an angiogenic response that leads to cancer metastasis and proliferation (David et al., 2016). Similar to our findings, higher plasma IL-8 levels have been associated with lower efficacy of anti-PD-L1 antibody (Yuen et al., 2020) in patients with UC. sTNFR-1 competes with the transmembrane form by binding to circulating TNFα, thereby inhibiting its action. Although TNFα is a pleiotropic cytokine with contradictory roles in oncoimmunology (Mercogliano et al., 2021), sTNFR-1-mediated inhibition of TNFα might suppress the anti-tumor activity of ICI in patients with UC. In addition, sTNFR-1 can reportedly increase IL-6 mRNA expression and stimulate IL-6 release (Wang and Bjorling, 2011), which may clarify the high correlation between plasma sTNFR-1 and IL-6 levels in the present study.

Herein, high baseline IL-12 (p40) and IFNγ levels were significantly associated with PD and worse OS and PFS, suggesting their importance as predictive markers. IFNγ is a well-known cytokine with anti-tumor immune effects, including activation and proliferation of tumor-infiltrating lymphocytes (Ikeda et al., 2002). Increased IFNγ levels after ICI treatment were strongly associated with better patient response and survival in non-small cell lung cancer (Boutsikou et al., 2018). In contrast, IFNγ has pro-tumor immune effects and plays a dual role in the tumor microenvironment. IFNγ promotes the expression of immunosuppressive molecules, such as indoleamine-2,3-dioxygenase 1 (IDO1), PD-L1, PD-L2, inducible nitric oxide synthase, Fas, and Fas ligand (Gocher et al., 2022). Furthermore, prolonged exposure to IFNγ signalling, associated with tumor growth, has been shown to promote these immunosuppressive molecules, resulting in resistance to ICI therapy (Benci et al., 2016). In the present study, the negative correlation between high IFNγ expression and OS and ICI response may be attributed to the pro-tumor immune effects of the IFNγ pathway. IL-12 (p40) is a cytokine and subunit of heterodimer IL-23. Both IL-12 (p40) and IL-23 are pro-tumor cytokines. IL-12 (p40) helps cancer cells escape cell death by suppressing the IFN-γ pathway and inhibiting cytotoxic T-cell infiltration (Kundu et al., 2017). IL-23 promotes IL-17 production and, like IL-6, activates STAT3 signalling. IL-17 promotes tumor angiogenesis and induces immunosuppressive molecules, such as tumor-associated macrophages and MDSCs, in the tumor microenvironment (Mirlekar and Pylayeva-Gupta, 2021). In renal cell carcinoma, IL-23 blockade augments the therapeutic effect of the anti-PD-1 antibody (Fu et al., 2019). The present study suggests the importance of the IFNγ pathway in ICI therapy, and further studies could identify potential strategies to enhance ICI treatment by inhibiting the pro-tumor effects of IFNγ and inducing its anti-tumor effects.

Changes in the post-treatment levels of IL-16, IL-22, IL-27, IL-28A, CCL21, CCL22, CXCL16, and osteocalcin were significantly associated with OS, similar to those of IL-6, IL-8, IL-12 (p40), and sTNF-R1. Elevated post-treatment levels of IL-27, CCL21, CCL22, and osteocalcin were associated with a favorable OS. These mediators have been reported to promote antitumor immune activity in the tumor microenvironment, and multiple studies have demonstrated their association with ICI therapy. For example, IL-27 directly stimulates CD8^+^ T cell differentiation and enhances antitumor cytotoxic T lymphocyte responses (Liu et al., 2013). An increase in IL-27 expression has been reported with anti-PD-L1 antibody treatment, leading to the suppression of tumor growth (Wenthe et al., 2022). CCL21 promotes the infiltration of T cells and dendritic cells into tumors, thereby enhancing immune activity (Sharma et al., 2020), and the synergistic tumor-suppressive effects of the combination of CCL21 and ICI in lung cancer and pancreatic cancer mouse models (Salehi-Rad et al., 2017; Chen et al., 2021). High expression levels of CCL22 and CCL17 are positively correlated with CD4^+^ T cell infiltration and a favorable prognosis with ICI treatment in patients with head and neck carcinoma (Zhou et al., 2022). Osteocalcin promotes T cell proliferation and IFNγ production, inhibiting tumor cell growth (Hayashi et al., 2017), and high expression levels of osteocalcin were associated with favorable OS in patients with head and neck carcinoma treated with the anti-PD-L1 antibody durvalumab (Arends et al., 2021). In contrast, elevated posttreatment levels of IL-16, IL-22, IL-28A, and CXCL16 were associated with poor OS. IL-22 has been identified as a poor prognostic factor in bladder cancer (Zeng et al., 2020). In addition, IL-22 has been shown to promote PD-L1 expression through the activation of STAT3 (Wolk et al., 2010; Chen et al., 2019), and the anti-PD-L1 antibody nivolumab has been reported to be effective in bladder tumors with a high density of intratumoral IL22-producing cells (Zeng et al., 2020). However, in this study, elevated post-treatment levels of IL-22 were associated with poor OS in the change analysis, suggesting that the function and immune evasion mechanisms of IL-22 differ between locations (intratumoral vs. peripheral), although further research is required. Furthermore, since no reports linking IL-16, IL-28A, and CXCL16 to ICIs have been found, further studies are needed.

Herein, low IL-6 and high TWEAK levels at baseline were significantly associated with irAEs. The mechanisms of irAEs remain poorly elucidated. However, irAEs have been associated with high plasma levels of proinflammatory cytokines in patients with melanoma patients (Lim et al., 2019). For example, high levels of IL-6, a typical inflammatory cytokine, are considered a risk factor for irAE, and tocilizumab, an anti-IL-6 receptor monoclonal antibody, can reportedly prevent irAEs in patients with melanoma undergoing ICI therapy (Dimitriou et al., 2021). However, the current study revealed that high IL-6 levels were negatively associated with irAEs. Given that there was an incubation period before the onset of irAEs and patients with UC exhibiting high IL6 levels had a markedly poor prognosis, disease progression may have occurred rapidly before the development of irAE. TWEAK signalling has been reported to activate inflammation and promote tumor invasion and growth (Winkles, 2008). Although it is speculated that TWEAK-mediated inflammation may be associated with the development of irAEs, no available reports have demonstrated an association between TWEAK levels and irAEs.

This study comprehensively and quantitatively surveyed soluble immune mediators in the plasma of aUC patients undergoing ICI treatment, and demonstrated associations of various immune mediators with immunotherapy response, and irAEs. These results indicate that interactions between multiple mediators may affect the tumor’s immunological microenvironment. However, this study had several limitations. First, the sample size was relatively small, which restricted the number of clinical factors included in the multivariate analysis and might limit the generalizability of the findings. Second, the uneven distribution of primary cancer sites and histologic types as well as the lack of standardized chemotherapy regimens prior to pembrolizumab treatment could potentially introduce bias. Third, the median follow-up duration was relatively short, mainly because of the rapid progression of aUC. Fourth, although many immune mediators have been shown to be associated with clinical outcomes, the clinical significance and predictive value of each mediator remain to be addressed in detail. Therefore, further large-scale studies with longer follow-up periods are warranted to provide a more comprehensive understanding of the patient outcomes. If further studies could establish immune mediators as predictive biomarkers of ICI response and irAEs, measurement of soluble immune mediators in plasma will be a promising strategy to guide treatment decisions and provide meaningful prognostic information, given the ease of monitoring over time and the reduced invasiveness that blood sampling offers.

## Data Availability

The raw data supporting the conclusion of this article will be made available by the authors, without undue reservation.

## Abbreviations

aUC, advanced urothelial carcinoma; CCL, C-C motif chemokine ligand; CR, complete response; CXCL, C-X-C motif chemokine ligand; ECOG PS, Eastern Cooperative Oncology Group performance status; IFN, interferon; IL, interleukin; irAE, immune-related adverse event; MDSCs, myeloid-derived suppressor cells; MIF, macrophage migration inhibitory factor; MMP, matrix metalloproteinase; OS, overall survival; PD, progressive disease; PD-1, programmed cell death protein 1; PD-L1, programmed death ligand 1; PFS, progression-free survival; PR, partial response; SD, stable disease; sTNF-R, soluble tumor necrosis factor receptor; TNF, tumor necrosis factor.
